# Efficacy of prophylactic intracameral antibiotic in preventing endophthalmitis after cataract surgery

**DOI:** 10.5935/0004-2749.2023-0345

**Published:** 2024-10-31

**Authors:** Constantin Philippe Salha, Lara Nascimento Machado, Myrna Serapião dos Santos

**Affiliations:** 1 Ophthalmology Department, Instituto de Assistência Médica ao Servidor Público Estadual, São Paulo, SP, Brazil

**Keywords:** Cataract extraction, Endophthalmitis, Antibiotic prophylaxis, Injections, Cefuroxime

## Abstract

**Purpose:**

To determine the impact of prophylactic intracameral cefuroxime administration on the post-cataract surgery endophthalmitis rates and analyze its safety.

**Methods:**

The incidence of post-phacoemulsification endophthalmitis before and after the introduction of antibiotic prophylaxis with cefuroxime was compared. Data were extracted from the electronic medical records of patients who underwent cataract surgery between July 2019 and July 2022 at a tertiary-care hospital. Data were also collected from the Hospital Infection Control Service database. Statistical analysis was performed to assess the efficacy of cefuroxime prophylaxis in reducing endophthalmitis rates.

**Results:**

Of the 4459 cataract surgeries included in the study, 2247 were included in the control group (pre-cefuroxime), and 2212 were included in the post-cefuroxime (ATB-P) Group. In the control group, 6 (0.13%) cases of endophthalmitis were reported. In the ATB-P Group, there were no cases of acute endophthalmitis. The frequency of endophthalmitis was significantly higher in the control group than in the ATB-P Group (p=0.016). Furthermore, *Staphylococcus* sp. was the most identified causative agent (75%). No adverse effects were reported after cefuroxime administration.

**Conclusion:**

The introduction of intracameral prophylaxis with cefuroxime significantly reduced the incidence of post-cataract surgery endophthalmitis. Additionally, its administration is safe.

## INTRODUCTION

Cataract surgery is the most commonly performed surgical procedure globally, with approximately 20 million eyes being operated on annually^(1)^. It is a highly safe procedure, with a postoperative complication rate of only 1.6%^(2)^. One of these complications is endophthalmitis. Although rare, with a varying incidence of 0.06% to 0.3%^(3)^, it could cause significant and irreversible visual loss.

Various methods have been studied to prevent postoperative endophthalmitis. However, the use of topical iodopovidone (5%) minutes before surgery is the only effective method of preventing endophthalmitis^(4)^. Topical antibiotics are widely used in the preoperative and postoperative periods of cataract surgery. However, robust scientific evidence regarding their effectiveness in preventing endophthalmitis is lacking^(4)^.

Intracameral injection of antibiotics was first introduced in the 1960s to treat different intraocular infections^(5^,^6)^. In recent years, several case series and retrospective studies have demonstrated a reduction in endophthalmitis rates with the routine use of intracameral antibiotics^(7^,^8)^. Nevertheless, there is still no consensus on the use of and class of intracameral antibiotics in the prophylaxis against postoperative endophthalmitis. Data from the 2014 survey by the American Society of Cataract and Refractive Surgery revealed that approximately 47% of cataract surgeons use or plan to use routine intracameral antibiotic prophylaxis^(9)^. In Europe, most surgeons use cefuroxime^(10)^. In Australia, vancomycin is the most used antibiotic^(10)^. Meanwhile, in India, moxifloxacin is commonly used^(10)^.

Despite being off label, intracameral administration of moxifloxacin has grown in recent years. In 2018, Lucena et al. conducted a prospective study in Brazil that demonstrated the safety of intracameral moxifloxacin^(11)^. Additionally, in 2019, the first randomized prospective study on intracameral moxifloxacin use was conducted in Brazil. The study revealed a sevenfold reduction in post-cataract endophthalmitis after the introduction of moxifloxacin^(12)^.

A prospective, multicenter, randomized study on the use of intracameral cefuroxime was conducted by the European Society of Cataract and Refractive Surgery (ESCRS). The study results revealed a fivefold reduction in the incidence of postoperative endophthalmitis^(13)^. Based on the increasing evidence in the literature^(14^-^16)^, prophylaxis with intracameral cefuroxime was implemented in April 2021 for all patients undergoing cataract surgery at the *Hospital do Servidor Público Estadual do Estado de São Paulo* (HSPE/IAMSPE).

In the present study, we aimed to evaluate the impact of introducing prophylactic intracameral administration of cefuroxime on the incidence of endophthalmitis in patients undergoing cataract surgery at the HSPE/IAMSPE. Additionally, we aimed to assess the occurrence of adverse effects following intracameral antibiotic admi-nistration and describe the etiology, treatments employed, and visual outcomes in patients who developed endophthalmitis.

## METHODS

This was a retrospective case series study. Data were extracted from the electronic medical records of patients who underwent cataract surgery between July 2019 and July 2022 at the Department of Ophthalmology at HSPE/IAMSPE. Additionally, data were collected from the Hospital Infection Control Service (SCIH - *Serviço de Controle de Infecção Hospitlar*) of IAMSPE. The study was approved by the Institutional Research Ethics Committee (CAAE: 53995321.7.0000.5463; December 29, 2021), and it was conducted in accordance with the principles of the Declaration of Helsinki.

Patients undergoing cataract surgery at HSPE/IAMSPE between July 2019 and July 2022 were included in the study. Patients lost during the early postoperative period, those with insufficient records, and patients diagnosed with toxic anterior segment syndrome were excluded from the study. The control group consisted of patients who underwent cataract surgery without intracameral prophylactic antibiotic administration, while the other group received intracameral prophylactic antibiotics.

Post-cataract surgery endophthalmitis was determined according to the criteria established by the National Health Surveillance Agency (ANVISA)^(17)^ (Table 1). The incidence of endophthalmitis before and after the introduction of intracameral antibiotic prophylaxis was compared, and the safety of the prophylaxis was analyzed.

**Table 1:** Diagnostic criteria for endophthalmitis

Criterion 1	Patient in whom microorganism was isolated from the vitreous humor via microbiological culture. OR
**Criterion 2**	Patient in whom an antimicrobial was injected intravitreally for the treatment of suspected endophthalmitis after another ophthalmic procedure. **OR**
**Criterion 3**	Patient with a medical diagnosis of endophthalmitis and the presence of two or more of the following signs and symptoms of ocular infection • Low visual acuity • Ocular pain • Corneal edema • Conjunctival hyperemia • Hypopyon • Anterior chamber reaction • Turbid vitreous

Available from: http://www.saude.sp.gov.br/resources/cve-centro-de-vigilancia-epidemiologica/areas-de-vigilancia/infeccao-hospitalar/2019/sve19_manual_endoftalmites_siven.pdf

All the patients were prepared for surgery in a standardized manner. The external periocular region was cleansed with 10.0% iodopovidone before surgery. Subsequently, the surgical site was disinfected with 5.0% povidone-iodine eye drops (PVPI) five minutes before the surgery. Cefuroxime was prepared by reconstituting an ampoule of cefuroxime (750 mg) with 15 mL of sterile water for injection. At the end of surgery, 1 mL of the reconstituted cefuroxime solution was aspirated and diluted in 4 mL of balanced saline solution. Thus, the resultant diluted solution contained 10 mg/mL of cefuroxime. Subsequently, 0.1 mL (1.0 mg) of cefuroxime was injected intracamerally at the end of the surgery. This protocol was validated by the SCIH of the IAMSPE.

The postoperative treatment protocol was standardized for all the surgeries. It included antibiotic eye drops (moxifloxacin) for the first week, and prednisolone eye drops for 30 days, which was gradually tapered.

The following data was collected from the records of patients who developed endophthalmitis during the study period: ability to isolate the causative agent in vitreous cultures, most isolated agent, treatment administered, and final visual outcome.

All data were analyzed using STATA (version 14.0; StataCorp LP, College Station, TX, USA). Frequency tables were used for descriptive analyses. The Fisher’s exact test was used to assess the difference in endophthalmitis frequencies between the two study groups. Statistical significance was set at p<0.05.

## RESULTS

Data of 4,459 cataract surgeries were included in the study. The control group comprised 2,247 surgeries performed between July 2019 and March 2021. The group that was administered intracameral antibiotic prophylaxis (ATB-P) consisted of 2,212 surgeries performed between April 2021 and July 2022.

During the study period, six patients developed endophthalmitis (frequency, 0.13%; 95% Confidence Interval (CI), 0.06-0.30%). All the cases of endophthalmitis were from the control group. Of the six cases, four were confirmed by a positive culture (frequency, 0.09%; 95% CI, 0.03-0.24%) (Table 2). The frequency of endophthalmitis was significantly higher in the control group than in the ATB-P Group (p=0.016). The organisms detected in the four positive cultures from the control group were *Staphylococcus lugdunensis* (50%), *Staphylococcus aureus* (25%), and gram-positive cocci + yeast (25%). The diagnostic features, treatments administered, and outcomes of the patients who developed endophthalmitis during the study period were presented in table 3.

**Table 2:** Surgical cases included in the study and the incidence of endophthalmitis

Study group	Total surgeries	Endophthalmitis cases	Positive culture	Estimated prevalence (%)	Confirmed culture prevalence (%)
Control group	2,247	6	4	0.27	0.18
ATB-P Group	2,212	0	0	0.00	0.00

ATB-P= intracameral antibiotics prophylaxis.

**Table 3:** Characteristics of the diagnostic time, treatment, and outcomes of the patients who developed endophthalmitis

	Group	Time until diagnosis (days)	Visual acuity at diagnosis	Treatment	Visual acuity post-treatment	Culture
Case 1	Control	7	HM	IIV Vanco + Ceftazidime and PPV	20/50	Positive
Case 2	Control	3	HM	IIV Vanco + Ceftazidime and PPV	20/40	Negative
Case 3	Control	10	HM	IIV Vanco + Ceftazidime and PPV	CF 1M	Negative
Case 4	Control	4	LP	IIV Vanco + Ceftazidime and PPV	CF 1M	Positive
Case 5	Control	5	LP	IIV Vanco + Ceftazidime and PPV	CF 2M	Positive
Case 6	Control	11	LP	IIV VANCO + Ceftazidime, PPV and VANCO + Ceftazidime	HM	Positive

HM= hand movement; LP= light perception; CF= counting fingers; IIV= intravitreal injection; PPV= pars plana vitrectomy; Vanco= vancomycin.

No adverse effects were reported in the study after the introduction of antibiotic prophylaxis with cefuroxime.

## DISCUSSION

The evaluation of prophylactic methods to reduce or prevent the incidence of endophthalmitis after cataract surgery has been the focus of several studies. The most suitable antibiotic for prevention should be a broad--spectrum antibiotic with minimal resistance that can be easily prepared and is safe for intraocular administration. Cefuroxime is a broad-spectrum antibiotic that covers most gram-positive and gram-negative organisms associated with postoperative endophthalmitis^(18^,^19)^.

The periocular flora is responsible for most of the post-cataract surgery endophthalmitis cases. This is because the local flora could enter the anterior chamber during or after the procedure and cause endophthalmitis^(20)^. The intracameral administration of an antibiotic at the end of surgery aims to eliminate any bacteria that may have entered and proliferated in the eye during the procedure^(20)^.

The effectiveness of intracameral cefuroxime in patients undergoing phacoemulsification has been demonstrated in recent years. The ESCRS’s randomized, multicenter, reference study revealed a fivefold reduction in the endophthalmitis risk in patients in whom intracameral cefuroxime was administered at a concentration of 1 mg/0.1 ml^(13)^. In another prospective study^(15)^ conducted in Spain, there was a 13-fold reduction in endophthalmitis risk (from 0.59% to 0.04%). In recent years, several retrospective studies have reached the same conclusion^(14^,^16)^. In 2016, the American Academy of Ophthalmology concluded that there is growing evidence that injecting an antibiotic intracamerally at the end of the surgery is an effective prophylaxis against endophthalmitis^(21)^.

In the present study, the incidence of endophthalmitis before the introduction of routine cefuroxime application was 0.27%, which was lower than the incidence in the ESCRS study (0.36%)^(13)^, the Au et al. study (0.43%)^(22)^, and the García-Sáenz et al. study (0.30%)^(15)^. However, it was higher than the incidence in the study conducted at Bascom Palmer Eye Institute (0.028%)^(23)^, in a large American study (0.11%)^(24)^, and in a study conducted in Hong Kong (0.11%)^(25)^.

After the introduction of routine cefuroxime application in 2,212 surgeries, there were no cases of post--phacoemulsification endophthalmitis (Table 2). Thus, the incidence decreased from 0.27% to 0%, which was a statistically significant result (p<0.05). This outcome is consistent with that obtained in the study by Ng et al. (0%)^(25)^. Although other studies also revealed a decrease in the incidence of postoperative endophthalmitis, the number of cases did not reduce to zero. In the classic ESCRS study^(13)^, prospective study in Spain^(15)^, and 2018 meta-analysis^(26)^, the incidence of postoperative endophthalmitis reduced to 0.05%, 0.043% ,0.03%, respectively, after routine antibiotic application. However, in a prospective study in India, there was no significant decrease in the postoperative endophthalmitis incidence (from 0.15 to 0.11%) after the introduction of intracameral cefuroxime application^(27)^.

As observed in the literature^(13^,^25^,^28)^, no adverse effects were reported after the introduction of cefuroxime in our study. A 2020 review determined that the main risk of adverse effects associated with cefuroxime application is related to its preparation^(10)^. Although there is increasingly strong evidence for its use, the lack of a commercially prepared single-dose cefuroxime for intracameral injection, such as that which exists in Europe, is a limitation reported by several ophthalmologists. The need for a two-step dilution in the operating room could be a possible risk factor for infection or more serious ocular damage^(10)^. However, even a dilution error (3 mg/0.1 ml) in the cefuroxime instilled in six patients did not cause any tissue damage, with good vision being achieved in all patients within a week^(29)^.

Among the patients who developed endophthalmitis in the control group, the diagnosis was confirmed by culture in 66.7% of the cases. This finding is similar to that of a study conducted in Barcelona (positive cultures, n=30, 65.8%) and lower than that of a study conducted in Hong Kong (87.5%)^(25)^. The most commonly isolated bacteria in our study was *Staphylococcus* (**Staphylococcus lugdunensis**, 50% and *Staphylococcus aureus*, 25%), accounting for 75% of the diagnosed cases. This finding is consistent with those of most studies that have concluded that *Staphylococcus* sp. is the main causative agent of endophthalmitis^(23^,^25^,^30)^.

In our study, the time to endophthalmitis diagnosis ranged from 3 to 11 days (average, 7 days) (Table 3). Furthermore, the visual acuity at the diagnosis was compromised in all the patients (hand motion, n=4; light perception, n=2). These findings are similar to those of the classic Endophthalmitis Vitrectomy Study^(30)^. At some point, all the patients with endophthalmitis were administered an intravitreal injection of vancomycin and ceftazidime, following which a by pars plana vitrectomy was performed^(23^,^30)^. However, even with the best therapy, most of the cases continued to evolve, resulting in a final low visual acuity (counting fingers, n=3; hand motion, n=1). This finding is consistent with those of several studies^(23^,^30)^.

A recent meta-analysis^(26)^ concluded that the use of postoperative antibiotic eye drops did not produce any significant reduction in endophthalmitis incidence when intracameral antibiotic had already been administered preoperatively. In developing countries like Brazil, intracameral antibiotic administration becomes crucial in preventing post-phacoemulsification endophthalmitis due to cataract surgery campaigns, risk of loss to follow-up in the postoperative period, and challenges faced by patients in understanding medical recommendations^(19)^.

This study has some limitations. Due to the retrospective nature of the study, there may have been a selection bias. Patients were grouped based on availability of historical data, which may not be a representation of the general population. Furthermore, the absence of randomization could have led to the formation of disparate groups. This may have complicated the establishment of a conclusive causal relationship. Despite these limitations, our study provides a preliminary finding, which needs to be validated through prospective and randomized studies.

The present study findings demonstrate that the introduction of intracameral prophylaxis with cefuroxime significantly reduced the incidence of post-cataract surgery endophthalmitis. Thus, intracameral administration of cefuroxime is safe. Furthermore, the microbiological characterization of endophthalmitis cases is crucial for determining the antibiotic that best fits the local microbiological reality.
